# Supplementary material for: Analysis of Lipid Metabolism in Adipose Tissue and Liver of Chinese Soft-Shelled Turtle Pelodiscus sinensis During Hibernation
Source: Int J Mol Sci. 2024 Nov 12;25(22):12124. doi: 10.3390/ijms252212124 (PMC11595087; doi:10.3390/ijms252212124)
Supplement: Supplementary file 1 [file ijms-25-12124-s001.zip › Supplementary Table.pdf]

Supplementary Table 1. Primers used for quantitative real-time PCR in this study

Gene	Primes (5'-3')	Product size (bp)
β-actin	F: GTGTTACCCATACTGTGCCCAT<br>R: AGCCATCTCCTGTTCAAAATCC	212
TPPP3	F: TTCCGCAAGTTCGCCATCTA<br>R: CAGTCCTTGCACAGTTTGGC	86
NAP1L4	F: GGGGTGCACAATTGAGTGGA<br>R: GCCTCGACCCTTGTGTTTCT	82
IL1R1	F: TCCTCTGAACAGCAACTGGC<br>R: CCATTTGATGACCCCCTGCT	147
DGAT2	F: GCATGAATGGGAAGAGGCTGT<br>R: ACAGTGCAAGCAACACCCAT	174
VGLL1	F: CCCTCACATGGTTCAGGGAC<br>R: GTTAGATGCTGGGCACCTGT	88
TANK	F: CAGGCATGCATGGACAGAGA<br>R: ATGGCCATGTGTGCTACCTC	174
MAOA	F: TGAGCGAGCTGTACGATGTG<br>R: TAAGCCCAGATTCAGCCAGC	84
RGS4	F: GAAGCATCGTCTGGGCTTCT<br>R: GCTGGACATCTTCGTGACCA	116
PRPF38A	F: ATTGGAGCCTCGGGTTAGTG<br>R: GTGCCCTATCCAGCTTGTCA	95

Gene	Primes (5'-3')	Product size (bp)
SORL1	F: TGGCCTGATCCGAGAGTACA<br>R: CCAGTTTCCTACACCCCGAC	163
PNPLA2	F: CCCTCTACATCCATCCTTCAA<br>R: AAATGCCCAGGCGTCCC	86
ABHD5	F: ATGGACGCTAACATTCTC<br>R: CTGTGGTCTACTACTACGC	169
LPL	F: ACTTTCGACATCCAGCGTGT<br>R: ATTTCACAAACACTGCCGCC	121
CPT1A	F: ATGAAGCCTCTATGACCC<br>R: TGTTTAGCAGCAGCAATC	154
PPARα	F: ACAGGTGAACAGGATGTGGAA<br>R: TCTCTGCCATACACAGCGTT<br>R: ATTTCACAAACACTGCCGCC	187
