# Supplementary figures and images for: Analysis of Lipid Metabolism in Adipose Tissue and Liver of Chinese Soft-Shelled Turtle Pelodiscus sinensis During Hibernation
Source: Int J Mol Sci. 2024 Nov 12;25(22):12124. doi: 10.3390/ijms252212124 (PMC11595087; doi:10.3390/ijms252212124)

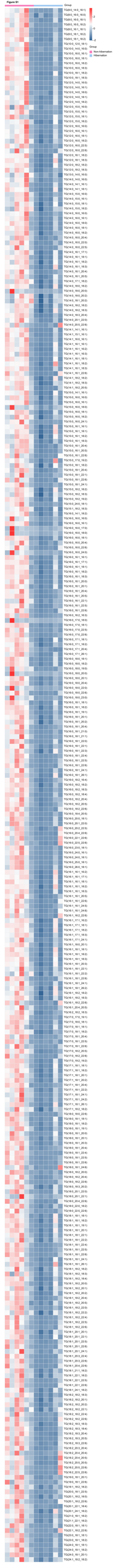

Figure S2

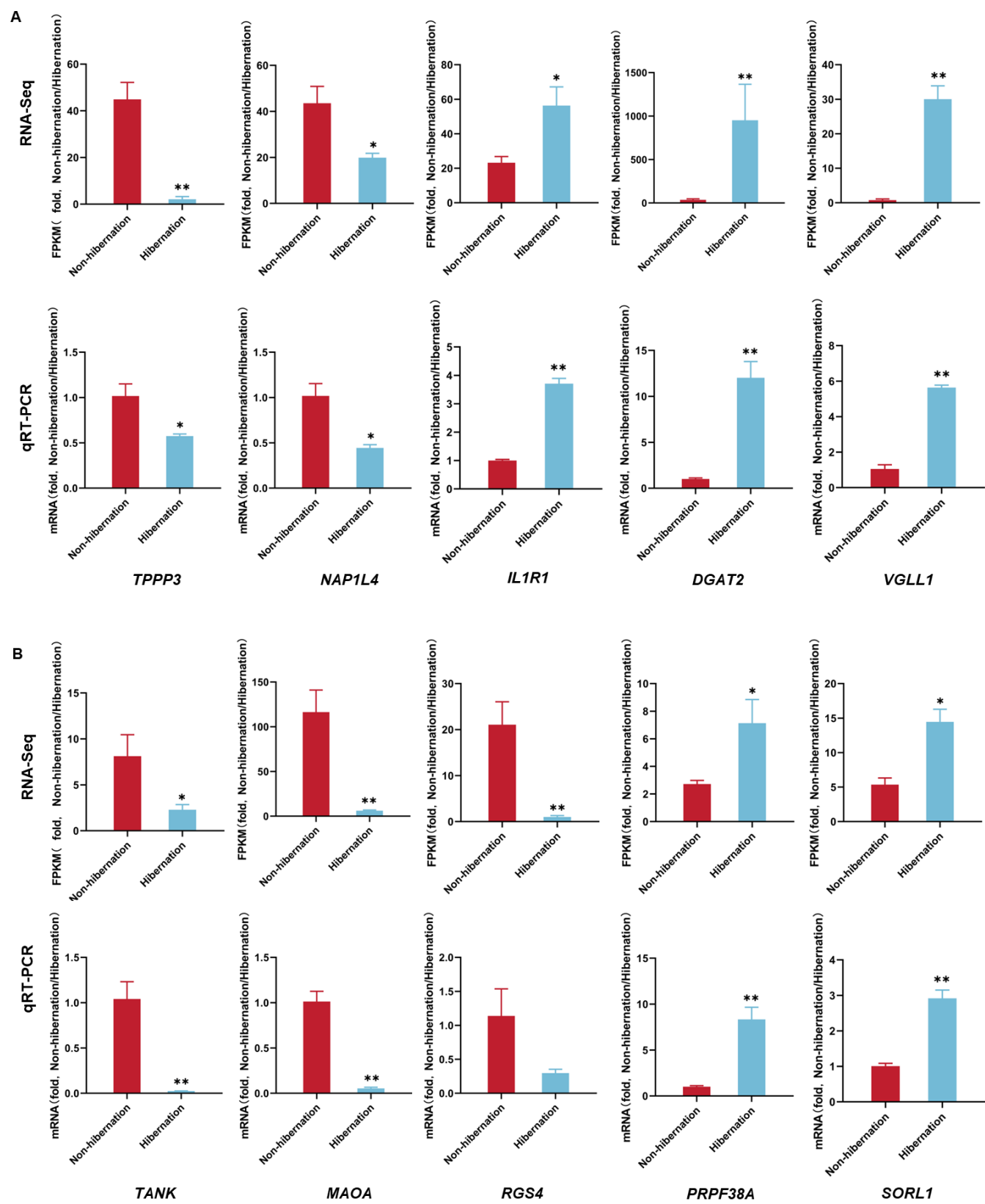

Supplement: Supplementary file 1 [file ijms-25-12124-s001.zip › Supplementary Figure.pdf]
